# Abstracts from the 10th International Conference for Healthcare and Medical Students (ICHAMS): Virtual. 12-13 February 2021

**DOI:** 10.1186/s12919-021-00209-4

**Published:** 2021-04-02

## PLENARY SESSION

### (Abstract A1)

#### A1. Scientific Analysis of MicroRNA Regulation of Risk Genes in Multiple Sclerosis (MS)

##### Giovanni Andrei Saw Ye Jeune, Chiara DeSanti, Claire McCoy

###### **Correspondence:** Giovanni Andrei Saw Ye Jeune

####### Royal College of Surgeons in Ireland, Dublin, Ireland

**Introduction**

Multiple Sclerosis (MS) is a chronic inflammatory disease characterised by the demyelination of the central nervous system in young adults. Although the cause for MS is unknown, genome-wide association studies have identified 233 genetic loci that are associated with MS susceptibility. The aim is to investigate whether these risk genes are predicted to be regulated by microRNAs (miRNAs) which are small non-coding RNAs involved in post-transcriptional regulation of gene expression.

**Methods**

200 non-MHC (Major Histocompatibility Complex) loci and 33 MHC-associated loci were interrogated to make a list of MS-risk genes. We utilised three microRNA target prediction algorithms, TargetScan, DIANA-microT-CDS and MiRDB, to find miRNAs that are most commonly regulated in that gene. Due to the limitation of the bioinformatic tool, we divided the MS risk genes into 7 distinctive categories based on their functions in molecular pathways and DNA regulation. Finally, a list of overrepresented microRNAs was compiled by intersecting the common miRNAs in each category and subsequently between those 7 categories.

**Results**

90 MS risk genes were selected from the 200 non-MHC loci and 11 genes from the 33 MHC-associated loci. We found that miR-27 and miR-4775 have intersections between 5 different categories, whereas miR-590, miR-548, miR-19, miR-3148, miR-340, and miR-153 were commonest in 4 different groups. A table compilation of all targeted MS risk genes with their most promising miRNAs and a Venn diagram representing these relationships were successfully generated.

**Discussion/Conclusion**

We were successful in the prediction search where miR-27 is commonly targeted in multiple pathways. Mir-27 is known to be potentially linked to immune regulation and this warrants an investigation on how altered miR-27 expression may regulate MS pathology. In conclusion, we have shown that microRNAs are predicted to target multiple MS-risk genes and that microRNA regulation may play a significant role in MS susceptibility.

## ORAL SESSION

### (Abstracts O1-O4)

#### O1. Medication, Nocturnal Dipping Profile and Hypertensive Emergency

##### Varahabhatla Vamsi^1^, Ingrid Prkacin^2^, Juraj Jug^3^, Martina Lovrić Benčić^4^

###### **Correspondence:** Varahabhatla Vamsi

####### ^1^King George Hospital, Visakhapatnam, India ^2^University of Zagreb, School of Medicine, Zagreb, Croatia; ^3^University of Zagreb, School of Medicine, Clinical Hospital Merkur, Zagreb, Croatia, ^4^University of Zagreb, School of Medicine, University Hospital Center, Zagreb, Croatia

**Introduction**

The aim of this study was to analyze the medication used by the patients with hypertensive crisis (blood pressure above 180/120mmHg) and its impact on the main risk factors for hypertensive emergency development.

**Methods**

A total of 233 patients (108 male, 125 female), 184 had hypertensive urgency/ 53 emergency (54.44% /50.95% in women) at the Emergency department during 11 months. Patients were divided in five age groups as decades starting from the age of 40 (mean 65.85 years) and a total ten groups depending on which type of hypertensive medication they were using (ACEi, ARB, BB, CCB, diuretics, moxonidine, and their combinations).

**Results**

By using antihypertensive monotherapy percentage of hypertensive emergencies were 100.00%, 50.00%, 41.66%, 33.33%, 21.05%. Using ACEi + CCB + diuretic significantly decreased the number of emergencies to 0%, 18.47%, 21.05%, 25.00%, 33.33%; but adding beta blocker additionally diminished the risk. Overall 53 patients had no medication (22.75%) and 68 of 233 patients were smokers (29.18%, 63.23% male) of which 36 patients had hypertensive emergency (52.94% of smokers). The biggest number of non-dippers was found in patients who took ARBs, diuretics and/or CCB but the smallest number was shown in patients who took ACEi in combination with moxonidine (-20.07%). 22.02% of smokers were non-dippers (-54.67% non-smokers). Odds ratio for getting hypertensive emergency in case patient had a non-dipper profile was 4.18 (Confidence Interval 1.02 – 18.89, p < 0.05). Patients taking different medication (or none) did not have an increased chance for hypertensive emergency development (Odds Ratio 1.21, p = Not Significant). We didn’t find any differences in the non-dipping profile incidence between genders (72.12% males, 72.83% females).

**Conclusion**

Combinations of all antihypertensive medication showed benefit over monotherapy. Higher 24-hour and nighttime blood pressure (non-dipping profile) was significantly associated with greater change for developing hypertensive emergency.

#### O2. YouTube videos on hands-only (compression-only) cardiopulmonary resuscitation: a content analysis

##### Reeya Gulve, Anuradha Joshi

###### **Correspondence:** Reeya Gulve

####### Bharati Vidyapeeth Deemed University Medical College, Pune, India

**Introduction**

YouTube videos are an important platform for sharing relevant healthcare information due to their wide accessibility, but the risk of disseminating misleading information should not be ignored. The purpose of the study was to evaluate the content accuracy of hands-only cardiopulmonary resuscitation (CPR) videos on YouTube according to the 2015 American Heart Association’s guidelines (AHA).

**Methods**

The YouTube was searched for videos in English using eight search terms related to hands-only CPR. First 60 videos of each search term were included in this study. Source of videos, total views, number of days since upload, likes, dislikes were noted. All the videos meeting the inclusion criteria were viewed and scored. Each step of hands-only CPR was given score as 0 (nonexistent or incorrect information) or 1 (sufficient information), and total score between 0 and 6 was assigned for each video.

**Result**

Out of 480 videos, 440 were excluded for variety of reasons and 40 videos were selected for study. Out of these 25 videos were uploaded by health organizations, health care institutes and hospitals, while 15 videos were uploaded by other sources. Mean content score of all videos assessed was 3.35. The mean content score was 3.40 for videos uploaded by health organizations, health care institutes and hospitals, while that for other sources it was 3.27. There was no statistically significant difference in views per day, likes, dislikes and content score among videos based on source.

**Discussion**

The results presented here showed that majority of hands-only CPR videos in English on YouTube on the study day were not compliance with the AHA guidelines in terms of providing basic information. Creation of high quality educational videos to be broadcasted is necessary, in order to adequately spread accurate and updated knowledge of hands-only CPR to the lay population.

#### O3. Virtual high-throughput docking study of approved drugs provides multiple, novel treatment options for COVID-19

##### Victoria Chan, Charlsea Maynard, Daire Fitzpatrick, Nathan Gnanasekaram, Maryam Khan, Anthony Chubb, Marian Brennan

###### **Correspondence:** Victoria Chan

####### Royal College of Surgeons in Ireland, Dublin, Ireland

**Introduction**

In November 2019, COVID-19 became a public health emergency of global concern. As the virus continues to spread rapidly, drug repurposing has gained some interest as possible treatment options. However, current drug candidates have been unsuccessful thus far at translating into the clinic, lacking therapeutic efficacy for patients. Aim: We aimed to use computational modelling to identify novel drugs for repurposing in COVID-19 patients.

**Methods**

We used virtual high-throughput screening to dock all the drugs that have entered clinical trials into two viral proteins: SARS-Cov2 RNA dependent DNA polymerase and the main protease (3CL^pro^ protease). *In vitro* data was then collected on these drugs for COVID-19 to identify their activity against COVID-19, C_max_ and toxicity.

**Results**

576 clinically tested drugs and drug candidates were predicted to interact with the COVID-19 targets. 97 candidates had reached phase I, 167 had reached phase II and 132 had reached phase III, 180 had reached phase IV. Of the approved drugs of interest, 15% were antivirals, 20% antibiotics, 4% antifungals and 1% antiparasitic drugs, that could be easily repurposed. Other interesting categories include the lung medications (4%), immunomodulating (4%) and antithrombotic/antiplatelet agents (4%) that may have positive effects on disease progression/presentation. The drugs predicted to have the best docking scores included drugs that have been shown to kill COVID-19 *in vitro* thus validating this modelling study.

**Conclusion**

We have utilised virtual high-throughput screening and data mining to identify a number of drug options that can be repurposed quickly and at scale either alone or in combination with other drugs. These drugs have the potential to be effective for use in the current pandemic.

#### O4. Investigating Macrophage Activation in Response to Damage-Associated Molecular Patterns in Multiple Sclerosis

##### Devika Dahiya, Caitlyn Loo, Jennifer Dowling, Claire McCoy

###### **Correspondence:** Devika Dahiya

####### ^1^Royal College of Surgeons in Ireland, Dublin, Ireland

Although the cause of Multiple Sclerosis (MS) is unknown, we understand that active macrophages release inflammatory mediators causing symptomatic damage. However, the trigger for macrophage activation is unclear. We investigated if the Damage Associated Molecular Pattern, High-mobility-group-box 1 (HMGB1), can trigger macrophage activation. HMGB1 is a ubiquitous nuclear architectural protein and was found to be upregulated in CSF samples and active plaques of MS patients. We measured the hallmarks of macrophage activation; NO (Greiss assay), IL-6, TNFa and IL-1b (ELISA), NFkB p65 phosphorylation (Western blotting), and miR-155 (RT-PCR). Raw 264.7 and bone marrow-derived macrophages were stimulated in a dose and time-dependent manner with HMGB1 and Toll-like receptor agonist, LPS, as a positive control. All experiments were performed in triplicate, 4 independent times. At 6, 24 and 48 hrs, LPS (1 mg/ml) induced activation markers (NO, IL-6, TNFa and IL-1b) in a time-dependent manner in both cell lines as expected. This was greatest with LPS at 48 hrs. HMGB1 alone (5, 10 and 50 ng/ml) did not have any impact on any of the activation parameters. However, when stimulated with both LPS and HMGB1, for 48 hrs, a statistically significant synergistic effect in NO, IL-6, TNFa and IL-1b production by Raw264.7 was seen. Our investigations imply that HMGB1 can synergistically enhance an inflammatory response in LPS stimulated macrophages. The impact of HMGB1 at later time points suggests it may worsen chronic inflammation, which may be relevant in MS patients who have increased HMGB1 and suffer chronic inflammation. Further characterisation will explore the different domains of the HMGB1 molecule on macrophage activation, as well as the mechanism of enhanced macrophage activation markers.

## POSTER SESSION

### (Abstracts P1-P42)

#### P1. A novel low-dose, intermittent gonadotropin-releasing hormone (GnRH) antagonist stimulation protocol for in-vitro fertilization (IVF)

##### Austin B. Auyeung^1^, Anthony Auyeung^2^

###### **Correspondence:** Austin B. Auyeung

####### ^1^Royal College of Surgeons in Ireland, Dublin, Ireland, Centre for Assisted Reproductive Services, Toronto, Canada; ^2^Centre for Assisted Reproductive Services, Toronto, Canada, Procrea Fertility Centre, Vaughan, Canada

**Introduction**

Follicle stimulating hormone (FSH) therapy used in IVF can produce premature luteinizing hormone (LH) surges and ovulation, both of which can be suppressed by GnRH antagonists (ANT). Standard ANT dosing is 0.25 mg daily from cycle day 5 or 6 until ovulation trigger. There are literature reports suggesting efficacy with lower daily GnRH ANT doses. This study was undertaken to determine if an IVF protocol using intermittent low-dose ANT can suppress premature LH surge and ovulation without compromising treatment outcomes.

**Methods**

In this retrospective chart review, IVF patients were stimulated with recombinant human FSH starting on cycle day 3. The first half-dose of ANT (0.125 mg cetrorelix) was administered between cycle days 6 to 8, and 2 to 3 days apart thereafter to maintain LH levels between 1.5 and 10 IU/L. Oocyte maturation and ovulation were triggered with recombinant human chorionic gonadotropin (rhCG) 36 hours prior to oocyte retrieval.

**Results**

Mean LH levels at baseline, prior to the second or third half-doses of ANT and just before rhCG trigger were 5.24±0.26, 7.83±0.88, 5.63±0.66 and 2.44±0.22 IU/L, respectively (n=50 cycles). An average of 14.58±0.95 oocytes were retrieved/cycle with 53% and 36% of the embryos continuing to cleavage and blastocyst stages, respectively (n=67 cycles). Of cycles that were completed to embryo transfer, 39% resulted in positive pregnancy tests with 32% persisting to clinical pregnancies (n=56 cycles). The average age of patients was 35.8±0.5 years. One percent (1%) of patients required one, 63% required two, 33% required three and 3% required four half-doses of ANT (n=67 cycles).

**Discussion**

Our study is the first to report an intermittent low-dose ANT IVF protocol using an average of 2.39±0.07 doses/cycle. Serum LH and premature ovulation were reliably suppressed in all cases, with no detrimental effects on oocyte, embryology or clinical outcomes.

#### P2. A Type 1 Diabetes Troubleshooting Guidebook in the Era of COVID-19

##### Jeneva Smith^1^, Manveer Singh^1^, Raymond Fung^2^, Zoe Lysy^2^, Rebecca Fine^2^

###### **Correspondence:** Jeneva Smith; Manveer Singh

####### ^1^Royal College of Surgeons in Ireland, Dublin, Ireland; ^2^Michael Garron Hospital, Toronto East Health Network, Toronto, Canada

**Background**

Type 1 diabetes is a complex, chronic disease; patients must be empowered and educated in their self-management in order to reach glycemic targets and decrease distress. This is essential to improving health outcomes and avoiding complications. However, many patients and health care providers do not know where to access reliable information regarding self-management. The aim of this research was to improve patients’ self-management of type 1 diabetes by developing an easily understandable guidebook, which provides comprehensive information on various situations that patients may encounter.

**Methods**

We used resources including clinical guides, journal articles and patient resources to create an outline for the guidebook. We solicited feedback from diabetes educators and endocrinologists, and conducted a focus group with patients to ensure the topics were relevant to them. Patient from the Michael Garron Hospital Type 1 Diabetes Support Group completed a survey about the content and usefulness of the guidebook. After incorporating the feedback, we wrote the guidebook using the gathered resources and it was assessed again. We then adapted the final guidebook to a website format.

**Results**

Over half of respondents to the survey indicated they would use the guidebook once a month or more. Every proposed topic was rated to be ‘helpful’ or better by at least 45% of people. The final guidebook contains 21 overall sections, such as how to deal with hypoglycemia and attending virtual visits.

**Discussion**

If properly utilised, the guidebook will serve as an educational and patient-centered intervention tool to improve people’s self-management of type 1 diabetes, thereby reducing negative health outcomes. This online tool is especially useful in the midst of the COVID-19 pandemic, when there is limited in-person access to physicians. Future research could examine if the use of the guidebook significantly improves health outcomes and seek patient feedback to improve the website.

#### P3. An Exploratory Study on The Relationship Between Self-Perceived Quality of Life and Physical Activity in a Canadian Cohort

##### Gerges Abdelsayed^1^, Joseph Girgis^1^, Nivin Azer^2^

###### **Correspondence:** Gerges Abdelsayed

####### ^1^Royal College of Surgeons in Ireland, Dublin, Ireland; ^2^Regent Primacy Medical Center, Winnipeg, Manitoba

**Introduction**

Physical activity has been linked to reduced risk of developing cardiovascular disease, stroke, diabetes, cancer, and depression. There is a positive relationship between physical activity and happiness. The aim of this explorative study is to assess the relationship between physical activity levels and self-perceived quality of life and self-satisfaction.

**Methods**

The survey was conducted in two medical clinics. It was created using combined, modified versions of the International Physical Activity Questionnaire and World Health Organization Quality of Life Questionnaire. This survey was comprised of two sections, including overall physical activity level and self-reported quality of life. The quality of life section required participants to rate their overall quality of life, health satisfaction, appearance satisfaction, and self satisfaction. Ethical approval was obtained. Valid consent was obtained, participants under the age of 18 were excluded. The data was analysed in STATA 16 using logistic regression, controlled for sex.

**Results**

The logistic regression model found a positive relationship between activity level and quality of life (OR: 2.31, p= 0.18), health satisfaction (OR: 2.36, p= 0.08 ), appearance satisfaction (OR: 1.78, p= 0.24 ), self-satisfaction (OR: 1.58, p= 0.32). None of these relationships were statistically significant, most likely due to the small sample size.

**Discussion**

This research did not find a statistically significant relationship. However, this does not mean that a relationship does not exist. This study had a small sample size, owing to its exploratory nature, meaning that levels of certainty required for statistical significance was not achieved. It is likely that if more participants are added to future replications of this study, then positive trends between activity levels and quality of life/satisfaction scores will become significant.

#### P4. An investigation of the potential growth promoting effects of marine-derived extracellular matrix components on neurons cultured in vitro

##### Fatima Alabandi^1^, Adrian Dervan^2^, Fergal O'Brien^3^

###### **Correspondence:** Fatima Alabandi

####### ^1^Royal College of Surgeons in Ireland, Dublin, Ireland; ^2^Advanced Materials and Bioengineering Research Centre (AMBER), RCSI and TCD, Dublin, Ireland; ^3^Tissue Engineering Research Group, Department of Anatomy and Regenerative Medicine, Royal College of Surgeons in Ireland, Dublin, Ireland

**Introduction**

The adult mammalian central nervous system (CNS) lacks intrinsic repair capacity. Following injury, astroglia secrete various chondroitin sulphate proteoglycan proteins (CSPGs), including 4-sulfated CSPGs (C4S) around the injury site that potently inhibit axonal regrowth. However, another secreted variant, 6-sulfated CSPGs (C6S) possesses growth-promoting properties, indicating that not all CSPG family may be growth inhibitory. In contrast to mammals, the fish CNS also contain CSPGs but are capable of regenerating their axons after injury throughout their adult lives, suggesting they may contain variants that promote axonal regrowth. The aim of this in vitro study was to screen CSPGs derived from 5 different fish species for axon-growth promoting properties.

**Methods**

Aqueous solutions of each CSPG diluted to 5μg/m, with poly-L-lysine as control, were used to coat sterile coverslips before being seeded with mouse motor neurons (NSC-34). After 7 days the neurons were fixed and stained with phalloidin-FITC and DAPI. Fluorescent images were acquired using a Nikon 90i microscope and analysed using the Neurite tracer plugin software for ImageJ.

**Results**

NSC34 neurons extended long neurites on all candidate substrates and exhibited a similar morphology compared to control. Quantification of mean neurite length revealed that neurons grown on CSPGs with the lowest C4S/C6S ratio had the longest neurite outgrowth, while groups with the higher C4S/C6S concentrations showed the lowest mean neurite outgrowth.

**Discussion**

These results suggest that neurons are capable of extending neurites when grown on marine CSPGs. They also indicate that C6S is conducive to stronger neurite outgrowth but also, those marine CSPGs with higher C6S concentration ratios are capable of negating the growth inhibitory effects of C4S. Fish CSPGs containing the lowest C4S/C6S concentration ratios properties could be engineered onto scaffolds to bridge CNS lesions and help support and direct axonal regrowth.

#### P5. Assessing Post-Operative Anxiety and Depression in Patients with Thyroid Pathology Amidst COVID-19

##### Vladimir Osadchyi^1^, William El Masri^2^, Bradley Hubbard^2^, Antoine Eskander^2^, Albino Chiodo^2^

###### **Correspondence:** Vladimir Osadchyi

####### ^1^Royal College of Surgeons in Ireland, Dublin, Ireland; ^2^Michael Garron Hospital, Toronto East Health Network, Toronto, Canada

**Introduction**

Due to the unprecedented nature of the COVID-19 pandemic, elective surgery around Canada has come to a screeching halt. This has caused surgery wait lists to greatly increase in size. Increased wait times can lead to negative psychological impacts on patients having to experience uncertainty and stress, during the pre-operative and post-operative time period.

**Methods**

Patients that were placed on the Michael Garron Hospital (MGH) thyroid surgery wait list were telephone interviewed and evaluated using the Hospital Anxiety and Depression Scale (HADS). HADS scores were analysed to assess psychological morbidity. Patients’ perspective on elective surgery postponement were obtained via a descriptive survey.

**Results**

A 36% response rate was achieved over a 3-week time period, with 16 patients completing the HADS tool and the descriptive survey. Mean HADS anxiety score was 7.86 and mean HADS depression score was 7.07, both of which fail to qualify as abnormal levels. 93.8% (15/16) participants indicated they experienced anxiety prior to having thyroid surgery. 62.5% (10/16) of participants indicated that increased communication with a healthcare provider before their surgery would reassure them of their health during the pandemic. 56.25% (9/16) of participants indicated having increased information regarding their surgery prior to the operation would reassure them regarding their health during the pandemic.

**Discussion**

Patients were found to experience no post-operative anxiety or depression according to the HADS tool. 93.8% of patients indicated they experienced anxiety prior to their operation. Patients indicated that increased communication with healthcare professionals and receiving more information regarding their surgery can help reassure them of their health. This can guide hospital administration’s actions and decision plans in the event of future elective surgery postponements, such as during another pandemic or a second wave of COVID-19.

#### P6. ASSOCIATION BETWEEN ELECTRONIC CIGARETTE USE AND ASTHMA SYMPTOMS AMONG ADOLESCENTS: A SYSTEMATIC REVIEW

##### Abdulaziz Alghanam^1^, Ali Ziyab^2^

###### **Correspondence:** Abdulaziz Alghanam

####### ^1^Royal College of Surgeons in Ireland, Dublin, Ireland; ^2^Health Sciences Center, Kuwait University, Kuwait City, Kuwait

**Introduction**

Electronic cigarettes (e-cigarettes) have gained substantial popularity among adolescents in recent years. The potential health effects of e-cigarette use are unclear.

**Objective**

The aim of this study was to systematically review observational studies that have investigated associations between e-cigarette use and asthma symptoms among adolescents.

**Methods**

A literature search of MEDLINE database through PubMed search engine was conducted for relevant observational studies. A total of 603 articles were identified and screened. Nine studies met the inclusion criteria and were included in this review.

**Results**

Most of the reviewed articles showed positive associations between e-cigarette use and asthma symptoms in adolescents. The odds ratio for the association between current e-cigarette use and asthma or wheeze were 1.12 (95% CI, 1.01-1.26), 1.48 (95% CI, 1.24-1.78), 1.34 (95% CI, 1.15-1.57), 1.78 (95% CI, 1.15-2.76), 1.86 (95% CI, 1.28-2.71) and 2.36 (95% CI, 1.89-2.94) in six of the nine studies.

**Conclusion**

Our review of the current literature indicates that e-cigarette use is positively associated with asthma symptoms based on results provided by cross-sectional studies. Hence, longitudinal studies are needed to corroborate the observed associations. Public health strategies are needed to raise awareness among adolescents on the potential harm of e-cigarette use and restrictions should be placed on e-cigarette sales and marketing to minors.

#### P7. BACTERIOPHAGES-BASED PREPARATIONS EFFICACY EVALUATION IN COMPLEX OF SUPPORTING THERAPY IN ONCOLOGICAL PATIENTS RECEIVING EGFR INHIBITORS

##### Elina Abdeeva^1^, Ekaterina Orlova^1^

###### **Correspondence:** Elina Abdeeva

####### ^1^I.M. Sechenov First Moscow State medical University (Sechenov University), Moscow, Russia

**Introduction**

According to modern data, the incidence of dermatological toxicity (DT) against the background of treatment of various cancers by inhibitors of tyrosine kinase receptors of epidermal growth factor (EGFR, epidermal growth factor receptor) reaches 90-95%. The most common methods of correcting acne-like manifestations of DT are the prescription of systemic antibiotic therapy, which is extremely undesirable for patients with metastatic liver damage. The aim of our study was a development of alternative methods for the prevention and treatment of acne-like manifestations of DT anti-EGFR therapy based on the study of skin microbiota composition.

**Methods**

The composition of the skin microbiota was evaluated by sowing the contents of the seedings on standardized environment at the time of inclusion and two weeks from the beginning of the therapy. The study included 24 patients in the standard therapy scheme (STS) and 20 patients in the "Phagoderm" therapy scheme (STSP). Statistical data processing was carried out using PASW Statistics 18. Level of reliability was defined as р ≤0.05 in all comparisons.

**Results**

In both groups, the localization of the voids on the face and upper torso 85% and 83% respectively. When comparing microbiota by qualitative composition, no significant difference in flora composition was detected. Both groups were dominated by Staphylococcus aureus and Escherichia Coli. Comparative evaluation of antibacterial therapy effectiveness on the 5th day showed 70% regression of spills in the STS group in 76% of patients, and 80% in the STSP group. In 33% (n-8) of the STS group, systemic antibiotics were cancelled due to an increase in SPGT and AST of 1.5-2 times.

**Discussion**

We assume that the inclusion of bacteriophage-based preparations in the maintenance therapy scheme is effective and can be used both as monotherapy in light severity manifestations and in complex therapy in intermediate and severe skin reactions.

#### P8. BREAST CANCER FEATURES IN NIGERIAN AND UKRAINIAN WOMEN

##### Shekinah Obinna, Jessica Otomara, Vladimir Porfirievich Shevchenko

###### **Correspondence:** Shekinah Obinna

####### Sumy State University, Sumy, Ukraine

**Introduction**

Breast Cancer(BC) is the most common malignancy in women of all races globally. A number of studies have suggested that there are differences between breast cancer among women In Europe and Africa. In Europe the incidence of BC is higher than in Africa but African women tend to die more from it. Studies on the features of BC among Nigerian and Ukrainian women have not been conducted.

**Aim**

To study the difference in BC between white Ukrainian and black Nigerian women.

**Materials and Methods**

Statistical date of hospital based studies in Nigeria and National cancer registry of Ukraine.

**Results**

The incidence of BC in Nigeria was -24.5 per 100,000 of population, which is significantly lower than in Ukraine-71.4, and in the Sumy region- 77.0. Mean age at presentation of BC varies between Nigeria and Ukraine, it is 48 years in Africa and two-third of the women are premenopausal. Delayed diagnostic of BC in Nigeria is related to lack of acceptance of orthodox treatment, low quality of medical care, local beliefs and ignorance of the disease. This is in contrast to Ukraine, where the system of cancer care for the population is developed, regular screening is available to women of certain ages. This increases the probability of detecting BC at a very early stage. As a result most women in Nigeria only receive palliative care because the BC is advanced and inoperable.

**Conclusion**

The Incidence of BC is low in Nigeria compared to the incidence in Ukraine (and in particular in the Sumy region). This may be due to illegality of abortion, high parity with prolonged breast feeding. In Nigerian women BC tends to present at an earlier age, but diagnosed in advanced stages and the results of treatment are unsatisfactory.

#### P9. Brewing Caregiver Burden: Cross-Sectional Study of Caregiver Burden in Alcohol Use Disorder

##### Vishnu Unnithan^1^, Kranti Kadam^2^

###### **Correspondence:** Vishnu Unnithan

####### ^1^Seth GS Medical College and KEM Hospital, Mumbai, India; ^2^Department of Psychiatry, Seth GS Medical College and KEM Hospital, Mumbai, India

**Introduction**

Alcohol use disorder is a growing problem. Families of these patients bear the burden of caring for them, feeling responsible for their relapse. The primary caregivers are at increased risk for stressful life events and psychiatric disorders. There is a gap in literature regarding sociodemographic variables of caregiver burden and its relation with variables of alcohol use disorder.

**Aims and Objectives**

The study assessed the sociodemographic profile of primary caregivers of patients diagnosed with alcohol use disorder as per DSM V and their severity of caregiver burden. The association and correlations between various variables of sociodemographic factors, alcohol usage and caregiver burden were also examined.

**Methods**

Cross-sectional observational study was carried out at a de-addiction centre attached to a tertiary hospital. Using Cochran’s formula and purposive sampling technique, primary caregivers of 80 patients were recruited over two months. Information was collected using structured questionnaire and Caregiver burden scale. Statistical analysis and Pearson’s correlation was done using GraphPad Prism.

**Results**

There is 78.75% prevalence of moderate to severe caregiver burden among the primary caregivers. There was a positive correlation between caregiver burden and quantity of alcohol consumed, monthly alcohol expenditure and years of marriage. The association between caregiver burden and various sociodemographic variables were not found to be statistically significant.

**Conclusion**

Alcohol use disorder can affect people of all socioeconomic classes irrespective of their education or employment status. Addressing the quantity of and expenditure on alcohol consumption with focus on caregiver psychoeducation will have significant implications in the rehabilitation of patients with alcohol use disorder. The motivation provided by caregivers is a strong determinant of the outcome of the rehabilitation process. Caregiver support groups can provide the missing spark that spurs patients to recovery.

#### P10. Can We Diagnose Thoracic Outlet Syndrome Faster with Neurophysiological Diagnostics?

##### Agata Kaczmarek, Anna Kalek, Juliusz Huber

###### **Correspondence:** Agata Kaczmarek

####### Department of Pathophysiology of Locomotor Organs, Poznan University of Medical Sciences, Poznan, Poland

**Introduction**

Thoracic Outlet Syndrome (TOS) is a group of conditions characterized by compression of the nerves, arteries and veins in the lower neck and upper chest area. On average, 6.5 physicians of different specialities need 4.3 years to develop TOS diagnosis. In our work, we would like to find a way to improve the diagnostic process.

**Methods**

Sixteen healthy subjects and 16 patients in the age between 18 to 36 took part in the study. Patients with clinically confirmed TOS were qualified from the outpatient clinic. In both groups, we performed neurophysiological studies such as the test of raised hands, Von Frey’s Filaments, electromyography (EMG), electroneurography (ENG), and motor evoked potentials (MEP).

**Results**

Sensory perception studies revealed changes in innervation more ulnar than the median nerve in the patient’s group. We also observed abnormalities in amplitudes of EMG recordings. ENG findings showed an axonal type of nerve injury. MEPs recordings, which were novum of our study, revealed the loss of efferent impulses transmission along the motors pathways for the cervical level to upper extremities effectors of “efferent block type”.

**Discussion**

Detection of TOS symptoms with neurophysiological tests in more than 50% of patients indicates the usefulness of complementary noninvasive diagnostics. Especially MEPs seem to be a fast and specific test for evaluating TOS symptoms regarding changes in motor impulses transmission.

#### P11. Characterizing the Tumour Immune Microenvironment in Early Breast Cancer

##### Alyssa Francis^1,2^, Luke McCaffrey^1^

###### **Correspondence:** Alyssa Francis

####### ^1^Rosalind and Morris Goodman Cancer Research Centre, Division of Experimental Medicine, Department of Oncology, McGill University, Montreal, Canada

^2^Royal College of Surgeons in Ireland, Dublin, Ireland

**Introduction**

Ductal carcinoma *in situ* (DCIS) is a neoplastic proliferation of epithelial cells confined to the luminal compartment of mammary ducts, which precedes invasive ductal carcinoma (IDC) formation. Only 20-50% of DCIS progresses to IDC, however it remains unclear what determines the likelihood of progression. We hypothesized that interactions between early tumour cells and the immune system leads to changes in the composition and activation state of the tumour immune microenvironment to become more suppressed, thereby contributing to the ability for DCIS to progress to IDC.

**Methods**

Using a panel of epithelial, immune and signalling markers, we characterized the tumour microenvironment of coexisting DCIS and IDC by imaging mass cytometry (IMC), across multiple patient samples, while controlling for inter-individual heterogeneity. Single-cell information was extracted and utilized to categorize cells and reconstruct spatial organization maps. Immune phenotype composition, cell-cell interactions, and tumour infiltrating lymphocytes (TILs) were evaluated, and nearest neighbour analysis was performed. Statistical analysis was done using ANOVA, Shannon's diversity index, and Kullback-Leibler Divergence.

**Results**

Although significant inter-patient heterogeneity was observed with respect to immune cell composition, overall, the activation state of the immune microenvironment indicated a suppressed phenotype was acquired with progression to IDC. This is evidenced by a significant increase in TILs as well as a decrease in the proportion of CD8 T cells to CD4 T cells observed in IDC tumours in comparison to DCIS tumours. There was also a significant increase in the tumour proliferation marker, Ki67, with progression to IDC. An increase in Treg:CD8 T cell and Treg:macrophage ratios was also observed, pointing towards a suppressed microenvironment, conducive to tumour outgrowth.

**Conclusion**

These results support our hypothesis that the immune microenvironment acquires a suppressed phenotype with DCIS progression and can serve to help guide future research investigating potential prognostic criteria for DCIS patients.

#### P12. Cochlear Implantation in Children with Asymmetric Hearing Loss

##### Urvashi Naraine^1^, Cristina Simoes-Franklin^1,2^, Laura Viani^1,2^, Christine Sheehan^2^, Olivia Ferguson^2^

###### **Correspondence:** Urvashi Naraine

####### ^1^School of Medicine, Royal College of Surgeons in Ireland, Dublin, Ireland; ^2^National Hearing Implant and Research Centre, Beaumont Hospital, Dublin, Ireland

**Introduction**

Cochlear implantation is a surgical procedure used to treat bilateral profound sensorineural hearing loss. In a population of 99 unilateral cochlear implanted paediatric patients, this study aimed to quantify their initial level of asymmetric hearing loss (pre-implantation). Also, the functional benefits of cochlear implantation are dependent on many factors, such as device usage compliance, environment, and pre-implant speech and language abilities. These variables were also considered to determine the benefits of cochlear implantation in this cohort of patients.

**Methods**

This audit reviewed the clinical charts, device parameters, and outcomes for each patient. This information was obtained via Beaumont Hospital National Hearing Implant and Research Centres’ records. Data from each child’s pre-operative auditory brainstem response (ABR) or audiogram for both ears classified the children according to their hearing loss asymmetry. Their cochlear implant’s device related details were analysed, and speech perception and intelligibility improvements were noted to see the functional benefits of cochlear implantation. This was done using the Category of Auditory Performance (CAP) and Speech Intelligibility Rating (SIR) scores.

**Results**

Patients were categorized into 1 of 3 groups based on their hearing loss asymmetry ranging from least to most severe. This was done using the variables decibel difference between the implanted and non-implanted ear and hearing loss ratio. The decibel differences for Groups 1 to 3 were, <10, 10-29.9, and ≥30, respectively and the ratio of hearing loss were, ≥0.9, 0.8-0.9, and <0.8, respectively. Additionally, after implantation, device related information showed no remarkable differences among groups, while all three groups experienced an improvement in both post-implantation CAP and SIR scores.

**Discussion**

Upon obtaining a scale to quantify each patient’s initial degree of hearing loss asymmetry, this study confirmed the functional benefits of cochlear implantation for all three groups of children with regards to hearing, speech, and language performance.

#### P13. COMPARATIVE STUDY OF VARIOUS METHODS OF FETAL WEIGHT ESTIMATION AT TERM PREGNANCY

##### Aashvi Patel^1^, Shivani Valia^2^

###### **Correspondence:** Aashvi Patel

####### ^1^GCS Medical College, Ahmedabad, Gujarat, India; ^2^Sumandeep Vidyapeeth University, Piparia, Waghodia, Vadodara, Gujarat, India.

**Introduction**

Accurate estimation of fetal weight helps in care of newborn and is an important parameter for perinatal morbidity and mortality.

**Methods**

Study design: prospective, non-interventional, comparative study. Selection criteria Inclusion criteria : All patients with singleton pregnancies with cephalic presentation who came at term with labour pains, for induction of labour or for elective full term LSCS with recent ultrasonography (within 1 week prior to delivery). Exclusion criteria: Abnormal pregnancy. Estimated fetal weight was calculated by: clinical methods - Dare’s and Johnson’s method. Sonographically by Hadlock’s formula. Estimated weights were compared with the actual birth weight.

**Results**

<2Kg, all methods could be used as there was no statistically significant differences between them. 2.0-2.5 & >3.5Kg,Hadlock’s formula was found to be better. 2.5-3.0Kg,Dare’s method correlated well with the actual fetal weight. 3.0-3.5Kg, Johnson’s formula correlated well.

**Discussion**

74% of the cases belonged to the group of 2.5-3.5 kg. Here, Dare’s method surpassed USG. Overall, USG was most accurate amongst the three methods. When the clinical methods were compared, Dare’s method was better than Johnson’s method. 60% of the cases were in the group of 2.5-3 kg, where the Dare’s method showed least average error for detecting the fetal weight. The estimated fetal weight was well correlated with birth weight with only 15% error in 93% cases in Dare’s method and USG, while in Johnson's method, same was true for 76% cases. Study conducted by BJ0110005, KLE University, in 95% of cases, Dare's formula and USG correlated well with actual birth weight with error of 15%. Both studies indicate that Johnson's formula lags behind in estimating fetal weight. USG was found to be most accurate method in both studies, when not available it could be replaced by Dare’s method as mean weight difference by the two methods was not statistically significant.

#### P14. COMPARISON BETWEEN FIRST-TIME AND REPEATED CESAREAN SECTION PLACENTAS

##### Loreta Miluna, Beatrise Gustsone

###### **Correspondence:** Loreta Miluna

####### Riga Stradins University School of Medicine, Riga, Latvia

**Introduction**

What exactly can we get from pathological interference for placental samples? What does it change in gynecological ward? This research target was to determine the differences of histopathology in first-time and repeated Cesarean section placentas delivered by women in 3^rd^ trimester and compare them.

**Methods**

This retrospective study included placental samples from 100 women with a singleton gestation who delivered at 3^rd^ trimester. Differences between placental samples as placental weight, inflammatory process and signs of vascular malperfusion of 80 first-time Cesarean section pregnancies and 20 samples of repeated SC pregnancies were analysed.

**Results**

65% (n=52) first-time SC placentas and 30% (n=6) repeated SC placentas had maternal inflammatory response. 3.75% (n=3) of first-time SC placentas and none of repeated SC placentas had fetal inflammatory response. 43% (n=34) first-time SC placentas and 25% (n=5) repeated SC placentas had decreased placental weight. 6.25% (n=5) placentas from first-time SC and 5% (n=1) from SC scar placental sample had hemorrhage. 37.5% (n=30) first-time SC placentas and 25% (n=5) repeated SC placentas had increased syncytial nodule index level. 45% (n=36) placentas from first-time SC and 25% (n=5) from SC scar placentas had fetal retardation.

**Discussion**

Signs of inflammatory process and uteroplacental blood malperfusion (UBM), such as - decreased placental weight, multiple placental infarctions and increased syncytial nodule index level are more common in first-time SC pregnancy. Although it may seem that repeated cesarean sections are much more dangerous to the health of the mother and fetus. On the one hand, this could indicate that repeated caesarean section does not increase the complication, but in order to evaluate it, we should additionally analyze all possible confounders in the future, e.g. hypertension that is likely to be more common.

#### P15. Co-overexpression of Tumour Necrosis Factor Alpha (TNFa), Junctional Adhesion Molecule-A (JAM-A) alpha(v)beta(3) integrin, RhoA and CD9 is associated with worse prognosis in breast cancer patients.

##### Faizah Abubakar Sani, Ann Hopkins

###### **Correspondence:** Faizah Abubakar Sani

####### Royal College of Surgeons, Dublin, Ireland

**Introduction**

Junctional Adhesion Molecule-A (JAM-A) belongs to the immunoglobulin superfamily of proteins and is expressed by epithelial cells, endothelial cells, neutrophils and platelets. Localising at intercellular tight junctions in epithelial and endothelial cells, JAM-A regulates adhesion. During neutrophil extravasation across endothelial cells, JAM-A is known to migrate to cell surface, making the endothelial cells less polar and aiding in extravasation. The migration of JAM-A is aided by TNFa expression, RhoA, alpha(v)beta(3) integrins and CD9. Since JAM-A over-expression has been linked with poor prognosis in breast cancer patients, the aim of this study was to determine if genes associated with JAM-A over-expression during extravasation are also linked to breast cancer.

**Methods**

An online tool (https://kmplot.com/) was used to test correlations between concurrent high mRNA levels of TNFa, RhoA, JAM-A, CD9 and alpha(v)beta(3) integrin and prognosis in a cohort of breast cancer patients.

**Results**

Patients(pt) in cohorts that were systemically treated with any endocrine therapy or chemotherapy who concurrently expressed high mRNA levels of JAM-A, TNFa, RhoA, CD9 and alpha(v)beta(3) integrin had a significantly poorer Relapse-Free Survival (RFS); with median survival of 185.16 months(1975pt) compared to those with lower levels (216.66 months)(1976pt) (n=3951 ?; p<0.0059). Subdivision of the patient cohorts according to estrogen receptor (ER) expression status revealed that in fact patients who were ER+ had better outcomes if they co-expressed lower levels of all genes (median survival of 68.71 months(1541pt) in ER+ compared to median survival of 54.96 months(1541pt); n=3082; p<0.033).

**Discussion**

Using publicly-available gene expression data from the Kaplan Meier plotter, we were able to show that concurrent over-expression of all genes is associated with worse outcomes in breast cancer patients overall and in ER+ patients. No correlations were visible for non ER+ patients due to lower number of patients.

#### P16. Correlation between physical activity & levels of depression and anxiety in Greek young adults. A cross-sectional study

##### Smaragda Skalidou^1^, Emmanouil Skalidis^1^, Andreas Anestis^1,2^, Nikolaos Papadakis^1^

###### **Correspondence:** Smaragda Skalidou

####### ^1^Laboratory of Hygiene, Social-Preventive Medicine and Medical Statistics, Department of Medicine, School of Health Sciences, Aristotle University of Thessaloniki, Thessaloniki, Greece; ^2^Division of Science and Technology, The American College of Thessaloniki, Thessaloniki, Greece

**Introduction**

Depression and anxiety disorders are among the underestimated threats for public health and especially the health of adolescents and young adults. Although physical activity is used in medical practice for treating these diseases, the number of studies investigating the correlation between exercise and mental illnesses is rather small. This study aimed to investigate the association between physical activity and the levels of depression and anxiety in Greek young adults.

**Methods**

A cross-sectional study was conducted on 268 adults (91 males) aged between 18 and 26 years. Data were obtained via online-administered questionnaires. Beck’s Depression Inventory (BDI) was used to assess the participants’ depression status. Hamilton’s Anxiety Rating Scale (HAM-A) was used to assess anxiety status. A chi-square test was performed and Pearson’s correlation coefficient was estimated for evaluating the association between categorical and continuous/ordinal variables, respectively. Student’s t-test and ANOVA were applied for comparisons between groups.

**Results**

Cronbach's coefficient α (0,755) indicated an acceptable level of internal consistency. Participants doing physical activities once per week or more, for at least 10-20 minutes, exhibited significantly lower levels of depression and anxiety (p=0,002 for BDI scores, p<0,001 for HAM-A score). Interestingly, although the frequency and duration of exercise did not show a significant correlation with the depression and anxiety status, a significant correlation of the latter with the participants’ perception of the overall significance of exercise was identified (Pearson’s r= -0,214 for BDI scores, p<0,001 and -0,190 for HAM-A score, p=0,002).

**Discussion**

Practicing physical activities seems to be correlated with lower depression and anxiety scores in the study population. Further investigation will allow additional modifiable (eg lifestyle) and non modifiable (eg gender, age) factors affecting the above association to be revealed.

#### P17. Developing a Virtual Community-Based Deprescribing Program

##### Margarita Rashev^1^, Meghan Abrahamson^2^, Tarek Dahche^2^, Justin Lin^2^, Andrew Liu^2^, John Abrahamson^3^

###### **Correspondence:** Margarita Rashev

####### ^1^Royal College of Surgeons in Ireland, Dublin, Ireland; ^2^Michael Garron Hospital, Toronto East Health Network, Toronto, Canada; ^3^University of Toronto Faculty of Medicine, Michael Garron Hospital, Toronto, Canada

**Introduction**

Inappropriate use of psychotropic medications is associated with a higher risk of adverse drug events (ADE). Evidence-based guidelines recommend gradual tapering and monitoring to mitigate withdrawal symptoms. Short-term inpatient stays are not amenable to the tapering of these medications. We will therefore use virtual care technologies to expand the scope of deprescribing at Michael Garron Hospital (MGH) (Toronto, Canada) and design and implement a hospital-based deprescribing program. Patients will be monitored ensuring their safety during the process of deprescribing of benzodiazepines, antipsychotics, and antidepressants.

**Methods**

Patients admitted to MGH taking psychotropic medications are identified using our electronic health record for a consult by a physician/pharmacist dyad. Patients/substitute decision makers are involved in a shared decision-making process consistent with their goals of care. Patients eligible and amenable will provide informed verbal consent to follow-up via virtual care mediums. Patients will be monitored over the course of their tapering schedule. Measured outcomes will include the proportion of patients who qualify for deprescribing, those who consent to virtual follow-up, and those who can successfully utilize this technology. We will measure the rate of complete cessation and dose reduction, and patient, primary care physician, and community pharmacist satisfaction with deprescribing. Balancing measures include patient barriers to virtual follow-up, ADE, and rehospitalization rates.

**Results**

We have designed an exhaustive protocol for this program which is under review from MGH Deprescribing Team members. We have also developed patient materials, including recruitment and post-consultation, drug-specific deprescribing information.

**Discussion**

Inappropriate use of these psychotropics is prevalent in older Canadians and older adults worldwide. This program will demonstrate the utility of virtual technologies in deprescribing initiatives for both the East Toronto community and beyond.

#### P18. Development of antisense oligonucleotide therapy for fibrodysplasia ossificans progressiva

##### Maria Mahfouz^1^, Rika Maruyama^2^, Toshifumi Yokota^2^

###### **Correspondence:** Maria Mahfouz

####### ^1^School of Medicine, Faculty of Medicine and Health Sciences, Royal College of Surgeons in Ireland, Dublin, Ireland; ^2^Department of Medical Genetics, Faculty of Medicine and Dentistry, University of Alberta, Edmonton, Alberta, Canada

Fibrodysplasia ossificans progressiva (FOP) is an autosomal dominant disorder characterized by progressive heterotopic ossifications (HO) where bone forms in skeletal muscle and soft tissues following trauma. HO is induced by inflammation and is cumulative, thus patients are immobile by their 20s. FOP is caused by a mutation (R206H) in the Activin A receptor type I (ACVR1) gene leading to a hyperactive receptor that induces HO outside of the skeletal system. Currently, there are no proven treatments for FOP, however reduction in ACVR1 gene expression is a promising therapeutic target. This can be established using antisense oligonucleotide (ASO) treatment which is a short deoxynucleotide strand designed to bind to target mRNA sequence and induce the mRNA degradation via Ribonuclease H1. Furthermore, the central portion of the deoxynucleotide monomers can be of a chimeric origin, named gapmer, that allows for allele-specific design. Four ASO treatments using 2' methoxyethyl (2'MOE) gapmers have been developed against the FOP ACVR1 mutation for a preferential knockdown of the FOP allele mRNA and thus reducing expression of the mutated ACVR1 receptor protein. In this project, the allele-specific knockdown effect of the FOP ACVR1 mRNA was tested *in vitro* using Human Embryonic Kidney derived clonal cells (HEK293T). Cells were transfected with either wild type or FOP ACVR1 DNA plasmids and treated with either a non-allele-specific/mock 2'MOE gapmer (used as a control) or with one of the four allele-specific 2’MOE gapmers. Protein was extracted from the transfected cells and measured using Western Blotting . Western Blot results showed an efficient allele-specific knockdown of the FOP ACVR1 allele compared to the wild type allele. Successful preferential knockdown of the FOP ACVR1 allele paves the way for in vivo testing and ultimately a new treatment agent for FOP patients that can eventually halt the progression of the disease.

#### P19. Efficacy and Safety of Esketamine as Treatment for Treatment-Resistant Depression: A Systematic Review and Meta-Analysis

##### Kevin Fernando^1^, Aruni Irfannadhira^1^, Yehezkiel George^1^

###### **Correspondence:** Kevin Fernando

####### ^1^Universitas Indonesia, Salemba, Indonesia

**Introduction**

Treatment-resistant depression (TRD) refers to inadequate response after taking at least two antidepressant drugs. Esketamine, an enantiomer of ketamine, has high affinity to NMDA. Nowadays, esketamine is widely used for TRD therapy, as an oral, intravenous, or intranasal drug. However, there was no review for efficacy and safety of esketamine as TRD treatment. Therefore, we conducted a systematic review to evaluate the efficacy and safety of esketamine in TRD patients.

**Methods**

Literature search was performed through databases Pubmed, ScienceDirect, CochraneLibrary, Wiley, Scopus, and ClinicalKey using (Treatment-resistant depression) AND esketamine AND efficacy AND safety as the keyword from inception to October 2020. Risk of bias was assessed with Cochrane ROB-2. The primary outcome was the change from baseline in Montgomery-Asberg Depression Rating Scale (MADRS) total point at the end of the treatment period. The secondary outcome was MADRS response rate, remission, and Treatment-Emergent Adverse Events (TEAE). A meta-analysis was conducted using the quantitative data.

**Results**

Six high-quality studies of RCT with a total of 474 patients were included. Esketamine was significantly more effective than placebo in reducing endpoint MADRS (MD=-6.11, 95%CI [2.95, 9.26], p=0.0001) and having higher odds ratios for rate of response (OR=1.94, 95%CI [1.07, 3.50], p=0.03). However, the remission rate between esketamine and placebo/ketamine showed insignificant difference (OR=1.69, 95%CI [0.97, 2.96], p=0.07). Patient undergoing esketamine therapy was associated with several TEAE, with statistical significant difference compared to placebo or ketamine in vertigo (OR=9.38, 95%CI [4.46, 19.73], p<0.0001), dissociation (OR=9.05, 95%CI [4.70, 17.41], p<0.0001), and increased blood pressure (OR=2.71, 95%CI [1.39, 5.28], p=0.003).

**Discussion**

In conclusion, we recommend esketamine as an adjuvant therapy for Treatment-Resistant Depression as it is considerably effective. However, several adverse events such as vertigo, dissociation, and increased blood pressure must be taken into consideration. Variation of dose and administration becomes the limitation of this study.

#### P20. Epigenetic Effects of Gemcitabine, Oxaliplatin, and Cetuximab

##### Olga Usalka^1,2^, Varvara Maksimova^1^, Guzel Sagitova^2^, Julia Makus^1,3^, Anastasia Patsyurkevich^1^, Valeria Popova^4^, Mariana Yakubovskaya^1^, Kirill Kirsanov^1,3^

###### **Correspondence:** Olga Usalka

####### ^1^N.N. Blokhin National medical Research Center of Oncology, Moscow, Russia; ^2^I.M. Sechenov First Moscow State medical University (Sechenov University), Moscow, Russia; ^3^RUDN University, Moscow, Russia, ^4^Moscow Polytechnic University, Moscow, Russia

**Introduction**

DNA methylation and histone modifications play a key role in the epigenetic regulation of gene expression. Aberrant changes in the levels of chromatin modifications affect the processes of proliferation, survival, adhesion, migration, etc. Previously we have shown that cetuximab, oxaliplatin, and gemcitabine activate the expression of the epigenetically repressed GFP gene in the HeLa TI test system. The aim of this study was to investigate the effect of these drugs on integral DNA methylation, level of histone methylation, activity of histone acetyltransferases (HAT).

**Methods**

The level of integral DNA methylation was analyzed by restriction analysis of genomic DNA with endonucleases *HpaII* (sensitive to unmethylated CCGG sequences) and *MspI* (sensitive to both methylated and unmethylated sites) and methyl-sensitive ELISA. Analysis of histone methylation levels was performed by Western blotting using antibodies to the H3K27me3 and H4K20me3 modifications. The activity of the HAT family enzymes was analyzed using HAT Activity Assay Kit.

**Results**

We showed that treatment of cells with cetuximab and gemcitabine did not affect total cytosine methylation. After oxaliplatin treatment a decrease in the fraction of DNA cleaved by methyl-sensitive endonuclease by 31% was shown by densitometry analysis of DNA electrophoresis, ELISA revealed the decrease of cytosine methylation by 18%, which indicates a small ability of oxaliplatin to demethylate DNA. There was an increase in the enzyme activity of HAT by 20% after oxaliplatin treatment. Changes in the levels of histone modifications H3K27me3 and H4K20me3 were not observed after analyzed drug treatment.

**Discussion**

Considering that oxaliplatin showed demethylating activity, as well as a tendency to increase the activity of histone acetyltransferases, it can be assumed that this drug is able to activate epigenetically repressed genes that should be considered when combined chemotherapy protocols are developed. Acknowledgements: This work was supported by the Russian Science Foundation (Grant No. 18-75-00115).

#### P21. Extent of Palliative Care need among cancer patients undergoing chemotherapy: A cross sectional study

##### Sai Prasad^1^, Jangala Sai Vihar^1^, Snehal Bathe^1^, Adithya Mohan^1^, Amlina Priyadarshini^1^, Lahari Boddu^1^, Rhea Singh^1^, Daanish Singh^1^, Yashaswi Sinha^1^, Vedant Jha^1^, Aishwarya Aiyer^1^, Jaiprakash Gurav^1^, Arshiya Duhan^1^, Aravind Chennath^1^, Tilak TVSVGK^2^

###### **Correspondence:** Sai Prasad

####### ^1^Armed Forces Medical College, Pune, India; ^2^Department of Internal Medicine, Armed Forces Medical College, Pune, India.

**Introduction**

Palliative Care is an interdisciplinary approach aimed at optimising quality of life and mitigating suffering. Identifying patients who may benefit from a palliative approach is a recognised challenge, especially in India, as it is associated mainly with end of life care. Therefore, an assessment of the extent of palliative care need in the hospital setting is crucial to appropriately match services and define priorities for care.

**Methods**

A cross sectional study was conducted among cancer patients undergoing chemotherapy (without adjunctive palliative care) at a tertiary care center in Western Maharashtra during February, 2020. After due consent, participants were screened for palliative care need according to the Gold Standards Framework (GSF) Prognostic Indicator criteria. Participants also completed the Sheffield Profile for Assessment and Referral to Care (SPARC), a needs assessment tool that measures unmet needs across 7 domains on a scale of 0-3. Data was entered in MS Excel and analysed using SPSS version 23.0.

**Results**

Out of 127 participants (mean age = 55.39 + 12.68), 38% met the GSF criteria for palliative care need. Patient self-reported data from the SPARC questionnaire indicated that participants who rated a score of 3 for >1 domains were 4.7 times more likely to meet the GSF criteria. The most frequently reported unmet needs were fatigue (76%), pain (71%), anxiety (49%), dependence (49%) and bowel and bladder issues (38%). Participants aged > 60 reported more concerns about loneliness (p=0.001) and anxiety (p=0.01), compared to younger age groups.

**Discussion**

Our results reveal that over a third of cancer patients undergoing chemotherapy met the GSF criteria for palliative care need. It provides evidence of a large unmet need across various domains among these patients, who may benefit from introduction of adjunctive palliative care, and lends support for the use of similar tools in the hospital setting.

#### P22. Financial And Psychosocial Impact of COVID 19: A Cross Sectional Community Based Study

##### Muhammad Aamir Anees^1^, Prajna Sharma^2^

###### **Correspondence:** Muhammad Aamir Anees

####### ^1^Kanachur Institute of Medical Sciences, Mangalore, India; ^2^Department of Community Medicine, Kanachur Institute of Medical Sciences, Mangalore, India

**Introduction**

In addition to continuous rise in cases and mortality due to Covid-19, lack of information and misinformation from unverified news sources has led to mental health crisis like anxiety and depression symptoms. The pandemic has also led to unemployment and decrease in family income. Hence in this study we intend to understand its financial and mental impact in the general population.

**Materials and Methodology**

A Cross sectional Community based study was conducted among the general population. Sample size of 100 was calculated and the study participants were selected using convenience sampling. Informed consent was taken before involving them in the study. A pretested validated anonymous online questionnaire was prepared and sent using google forms to collect information regarding their socio-demographic variables, financial status and the effect of media on their daily life. Patient Health Questionnaire -9 (PHQ-9) was used to assess depression among them. Data was analyzed using proportions, percentages, Independent sample t test and and statistical significance was set at p < 0.05.

**Results**

Of the participants who reported a drastic decrease in family income 19% mentioned that the pandemic has affected the availability of basic resources like food and water, 27% reported that a family member has lost a job during the pandemic. According to PHQ-9 scaling, 7% of the total participants were severely depressed, while 9% had moderately severe depression, 21% were suffering from moderate depression, 36% had mild depression and 27% had no symptoms of depression. Drastic reduction in family income was found to be responsible for higher PHQ9 scoring and was statistically significant (p< 0.05).

**Discussion**

The pandemic has had a significant financial and psychosocial impact on society. We recommend that Governments around the world to create an emergency fund for an unprecedented situation like this.

#### P23. HEALTH SEEKING BEHAVIOR AND LIFESTYLE OF PEOPLE WITH COMORBIDITIES DURING COVID-19 PANDEMIC: A PILOT STUDY

##### Megha Joy, Anilbindu S

###### **Correspondence:** Megha Joy

####### Sree Gokulam Medical College and Research Foundation, Venjarammoodu, Kerala, India

**Introduction**

COVID 19 has dramatically changed how outpatient care and follow-ups are delivered to comorbid population(DM, HTN,CAD).Fear of COVID infection have kept patients away from hospitals despite their need for follow-ups which could have poor clinical outcomes. Thus the research question:

- How did health seeking behavior and lifestyle of comorbid population change during COVID pandemic?

**Methods**

A quantitative cross sectional study based on non-random convenience sampling through telephonic interviews among adults with chronic diseases attending OPDs, followed by filling of semi-structured questionnaire in google forms.

**Independent variables:** age, comorbidity, gender, educational level

**Dependent variables:** Health seeking behavior, lifestyle practice

**Statistics:** Categorical data: frequency, percentage

Test of significance: Chi square test

P<0.05 – significant

**Results**

Among a total of 44 people (average age : 64.6), taken part in the study, 63.6%was with DM, 43.2% HTN, 15.9% CAD, 2.3% cancer, 2.3% chronic liver disease and the rest with other conditions for which have been taking treatments for at least 2 years.

50% of the study participants had regular follow ups scheduled among which 43.2% had faced delays since the COVID spread , 38.6% followed the same medication since then. There has been an increase of 18.2% in telemedicine use among the population compared to null use in the pre-COVID era. This can be attributed to the fear of getting infected by COVID19 as reported by 86.2% participants. A decrease of 6.3% in physical activity has been identified.Food habits remained more or less same.

**Conclusion**

The fear of COVID infection have made people avoid hospital visits at any costs. The practice of attending clinicians physically, have reduced greatly and got replaced by telemedicine which had not been a practice before the pandemic. A slight decrease in physical activity is seen in these population which may make them more vulnerable.

#### P24. Health service use and associated costs attributable to diabetes in the Mitchelstown Cohort Study

##### Patrick Walsh^1^, Kate O'Neill^2^, Patricia Kearney^2^

###### **Correspondence:** Patrick Walsh

####### ^1^School of Medicine, University College Cork; ^2^School of Public Health, University College Cork

**Introduction**

The number of people with diabetes is increasing globally and with evidence of rising medical expenditure per person, the growth in economic burden will continue. Accurate cost of illness estimates are needed to inform national policy and identify potential cost savings.

**Aim**

To estimate health service use and costs attributable to diabetes

**Methods**

A sample of middle-aged adults (≥ 50 years) from the Mitchelstown Cohort Study, collected between 2016-2017 was analysed**.** Diabetes was defined using self-report doctor-diagnosis, HbA1c and fasting plasma glucose levels. Health service use in the previous 12-months included; number of general practitioner (GP) visits, emergency department visits, hospital admissions, outpatient visits, and day procedures. Multivariable negative binomial regression was used to estimate the association between diabetes and frequency of visits. Frequency of visits was applied to unit costs for each health service, calculating mean costs per person with and without diabetes.

**Results**

Of 1,332 patients analysed, prevalence of diabetes was 10.4% (95%CI:8.9,12.2) [Diagnosed 7.4% (95%CI:6.1, 8.9), Undiagnosed 3.1% (95%CI:2.3,4.2)]. Diabetes was associated with a 49% increase in GP visits. Diabetes was not associated with additional hospital admissions, emergency department visits, outpatient visits or day procedures. The annual mean cost of health service use among those with diabetes was €1,597.80 per person compared with €1,352.67 for those without.

**Conclusion**

While diabetes was associated with additional GP visits, it was not associated with additional service use in secondary care. Structured diabetes management in primary care may contribute to reduced health service use and costs attributable to diabetes.

#### P25. Histomorphometric changes of lung lymphoid follicles in young rats under experimental alloxan hyperglycemia

##### Toufik Abdul-Rahman, Andrew Awuah Wireko, Tetiana Teslyk

###### **Correspondence:** Toufik Abdul-Rahman

####### Sumy State University, Sumy, Ukraine

**Introduction**

Diabetes has been and remains a global problem today, leading to disability, disability and death.

**Methods**

The studies were performed on 48 white laboratory rats of both sexes. Experimental animals were divided into two series: experimental and intact. Each experimental group is divided into subgroups: the first - with a term of hyperglycemia of 30 days, the second - 60 days, the third - 90 days, the fourth - 120 days. For experimental simulations of hyperglycemia, alloxan monohydrate was used. The perimeter of the lymphoid follicles (PLF) was measured. The level of glucose and glycosylated hemoglobin HbA1c in the venous blood of rats was determined before each slaughter of animals.

**Results**

The level of glucose in the blood of experimental animals from 30 to 120 days ranged from 13.3 ± 0.1 to 19.3 ± 0.2 mmol / l, from the end of the second month of the experiment, the level of HbA1C ranged from 7.1 ± 0 , 05 to 8.6 ± 0.08. In the intact group, the level of glucose in the blood was within normal limits. On the 30th day, the PLF in intact and experimental animals was 449.3 ± 0.82 μm and 449, ± 0.17 μm, respectively. From 60 days, hypertrophy of pulmonary lymphoid follicles with pronounced vascularization was noted, in comparison with intact animals, the PLF index was 2.3 times higher. Involute changes in lymphoid follicles and malnutrition were observed in intact animals at day 90. In experimental animals of the same age , PLF increased 1.3 times compared with the 60th day. On day 120 of the experiment, PLF in experimental animals increase by 3.8 times compared with intact animals.

**Discussion**

Against the background of chronic experimental hyperglycemia, young animals developed hypervascularization and hypertrophy of pulmonary lymphoid follicles, thereby causing obstruction in the lower respiratory tract.

#### P26. IL-6 synthesis by HeLa cancer cells. Synthesis modulation by polyphenols (Taxifolin as an example)

##### Albina Zagidullina, Vladimir Rogovsky

###### **Correspondence:** Albina Zagidullina

####### Pirogov Russian National Research Medical University, Moscow, Russia

**Introduction**

Cancer is one of the leading causes of death of people worldwide. Researchers around the world try to find different ways to impede tumor growth and cancer progression, one of the latest news in this area is the possibility of natural polyphenols to prevent and treat cancer. In our research, we decided to check the possible action of polyphenols such as Taxifolin on the immune microenvironment of the cancer cells.

**Materials**

For this purpose, we used HeLa cells, which were cultivated in the culture media (Bovine Serum + DMEM). Then we placed 10000 cells in each well of the plate. The supernatant was taken from each well for the ELISA procedure. ELISA was used for the IL-6 determination in the cell culture. The analysis was provided with the usage of GraphPad Prism.

**Results**

There was a relatively high secretion of IL-6 by HeLa cells (271 pg/ml in culture media). Taxifolin raised the concentration of IL-6 in culture media from 271 ± 11,5 pg/ml to 301 ± 18 pg/ml.

**Discussion**

On this step of our research, we get intermediate results which showed that the presence of natural polyphenol in the cell culture rises the production of IL-6 which plays an important role in tumor suppression. The increasing concentration of IL-6 can stimulate an inflammatory response and can lead to the domination of the tumor immune rejection processes over tolerance. However, the research work still proceeds, the comparison with other polyphenols (Curcumin, EGCG, Resveratrol) will be done for the more precise evaluation of the polyphenols' role in cancer treatment and prevention possibilities.

#### P27. Improved cookstove interventions to reduce household and ambient air pollution among the global poorest communities: A scoping review

##### Jessica Langevin^1^, Megan Davis^1^, Eunice Phillip^1^, Nitya Kumar^2^, Vincent Jumbe^3^, Mike Clifford^4^, Aisling Walsh^1^, Sarah Jewitt^4^, Joseph Mfutso-Bengo^3^, Maria Beard^4^, Debbi Stanistreet^1^

###### **Correspondence:** Jessica Langevin

####### ^1^Royal College of Surgeons in Ireland, Dublin, Ireland; ^2^Royal College of Surgeons of Ireland-Bahrain, Busaiteen, Bahrain; ^3^University of Malawi, Zomba, Malawi, ^4^University of Nottingham, Nottingham, United Kingdom

**Introduction**

Each year, the combined effects of household and ambient air pollution (HAAP) lead to approximately seven million premature deaths globally and contributes to a number of cardiovascular and respiratory diseases. The burden is highest in low and middle-income countries, with the major source of (HAAP) being combustion of fossil fuels for cooking and lighting. Despite the anticipated increase in access to clean cooking over the next twenty years, the absolute numbers of those in Africa who do not have access to clean fuels is expected to increase. Recognizing that these communities are unlikely to be able to afford the more expensive cooking technologies, it is important to identify affordable stove interventions that could reduce the impact of HAAP among the poorest communities.

**Objectives**

To explore evidence in relation to the cookstove-intervention options available to the global poorest communities to reduce HAAP, the characteristics (e.g. type, cost, availability), and health impact of these interventions.

**Methods**

This review followed the Joanna Briggs Institute’s framework. All biomass stove intervention studies conducted from 2014 to date, in Africa, and in English were included. Four reviewers conducted preliminary screening, followed by a full-text screening; 20% being double screened with uncertainties being resolved through discussion. Data extraction was completed for the remaining studies by all.

**Results**

Evidence in relation to stove type, efficiency, emissions, safety and price based on the Clean Cooking Catalogue data was recorded and compared with field results where available. Reduction in HAP and health outcomes were summarized. Where data were available, we also report availability and accessibility. The quality of included studies were assessed.

**Conclusion**

The outcome of this review, along with additional evidence from the literature, will be used to develop a tool kit guide to HAAP interventions for the poorest communities globally.

#### P28. Incidence of Early Complications After Modified Radical Mastectomy for Breast Cancer

##### Maryam Ehtesham^1^, Talal Almas^1^, Absam Akbar^2^, Muhammad Kashif Khan^3^

###### **Correspondence:** Maryam Ehtesham

####### ^1^Royal College of Surgeons in Ireland, Dublin, Ireland; ^2^Aga Khan University, Karachi, Sindh, Pakistan; ^3^Surgical Oncology, Maroof International Hospital, Islamabad, Islamabad Capital Territory, Pakistan

**Introduction**

Modified radical mastectomy is the primary surgical treatment for breast cancer. It is often associated with seroma formation postoperatively. Our study aims to address the possibility of this complication along with the likelihood of developing wound infection postoperatively.

**Methods**

Patients who met the selection criteria where identified and randomly selected. The age of patients, stage of breast cancer and the complications observed after six weeks were recorded. The data collected was then input and analysed using the SPSS version 21.0.

**Results**

Of the 65 patients selected, 3 patients (4.6%) developed a wound infection and 19 patients (29.23%) had seroma formation. An additional 18 patients (27.69%) with stage II breast cancer had seroma formation compared to merely 1 patient (1.54%) with stage I breast cancer. Furthermore, 3 patients with stage II breast cancer developed a wound infection while none of the patients with stage I breast cancer developed this complication.

**Discussion**

Modified radical mastectomy is an invasive surgical procedure that can elicit a myriad of complications, including seroma formation and wound infection. The study’s findings were consistent with national studies with regards to the incidence of seroma formation in the aftermath of modified radical mastectomy. Nevertheless, studies with larger sample sizes and better control-arms are needed in order to determine the true incidence of postoperative complications.

#### P29. Induced Pluripotent Stem Cells in the Potential Treatment of Type 1 Diabetes

##### Ryan Ahn^1^, Nidheesh Dadheech^2^, AM James Shapiro^2^

###### **Correspondence:** Ryan Ahn

####### ^1^Royal College of Surgeons in Ireland, Dublin, Ireland; ^2^Department of Surgery, University of Alberta, Edmonton, Canada

**Introduction**

Type 1 diabetes mellitus is a metabolic disease characterized by autoimmune destruction of pancreatic islets, causing a lifelong insulin deficiency. Insulin remains the mainstay of treatment for type 1 diabetes, but there have been tremendous strides in cell replacement therapy via allogeneic whole pancreas and islet transplantation. These methods are limited due to scarcity of tissue and the requirement of chronic immunosuppression. Induced pluripotent stem cells (iPSCs) have the ability to regenerate and differentiate into specialized cells due to genetic reprogramming with the Yamanaka factors (Oct3/4, Sox2, c-Myc, Klf4).

**Methods**

Peripheral blood mononuclear cells (PBMCs) were taken from a patient with surgically-induced diabetes. These cells were cultured for expansion and then reprogrammed into human iPSCs by introduction of the Yamanaka factors via a retroviral vector (Sendai virus). The iPSCs were expanded for 8 passages before they were tested for pluripotency by genetic analysis and immunofluorescence.

**Results**

Genetic analysis was performed via RT-PCR followed by gel electrophoresis, demonstrating the presence of Klf4, Oct4, Sox2, and c-Myc. However, this also confirmed the persistence of genetic material from the viral vector. Immunofluorescence verified the presence of Oct4 and Sox2, while also proving the expression of other pluripotency markers (Nanog, SSEA4, TRA-1-60, TRA-1-81).

**Discussion**

Characterization of the cells confirmed that the newly created iPSCs were truly pluripotent. Differentiation of these cells could yield functional patient-specific specialized cells, including functioning pancreatic beta cells. Tissue engineering with iPSCs could potentially have future applications in the treatment and possible cure of type 1 diabetes.

#### P30. Inter-rater variability for manual feature selection in retinal vascular optical coherence tomography imaging

##### Tiffany Yeretsian^1^, Yu Li^2^, Joel Ramjist^2^, Jillian Cardinell^2^, Nhu Nguyen^2^, Yuta Dobashi^3^, Chaoliang Chen^2^, Victor Yang^2^

###### **Correspondence:** Tiffany Yeretsian

####### ^1^Royal College of Surgeons in Ireland, Dublin, Ireland; ^2^Ryerson University, Toronto, Canada, ^3^University of Toronto, Toronto Canada

**Introduction**

The movement towards quantitative diagnostic biomarkers in medical imaging has shown increasing promise in disease identification and early detection. This is particularly true in more mature imaging modalities, including in Optical Coherence Tomography Angiography (OCT-A). OCT-A imaging can provide vital information regarding the state and morphological features of vascular networks, and particularly, has been used regularly in the examination of retinal vascular networks, with potential implications as a direct monitoring window into cerebrovascular networks.

**Methods**

In this preliminary study, 7 individual raters familiar with retinal OCT-A imaging are presented with a series of 40 images where maximal vessel diameter, and the basis evaluations for branchpoint count and tortuosity are determined through semi-automated or manual inputs, and variability of the recorded measures between raters are examined.

**Results**

The preliminary results demonstrate the variability that one may expect in a standard clinical dataset, and a potential hurdle that may need to be overcome when determining ground truths for training data in supervised neural networks.

**Discussion**

Features such as vascular fraction, fractal dimension, branchpoint count, tortuosity, and maximal vessel diameter hold particular promise as important biomarkers of interest; however, many of the current methods for determining these metrics are semi-automated or manual, requiring user judgement and input. Thus, the subjective nature of the user input may result in significant inter-rater variability, potentially causing quantitative evaluation to be left too variable for proper diagnostic accuracy; however, this has not been demonstrated. Moreover, with the emergence of new automated methods that could be employed, particularly with the use of machine learning, and understanding of this variance is key, especially with regards to supervised learning methods where ground-truths may be determined from rater data.

#### P31. Investigating the genetic changes in macrophages mediated by Il-10

##### Therese Lynn, Remsha Afzal, Claire McCoy

###### **Correspondence:** Therese Lynn

####### Royal College of Surgeons in Ireland, Dublin, Ireland

Macrophages play an essential role in the regulation of inflammation. In persistent inflammation however, macrophages may cause severe tissue damage and are implicated in a number of inflammatory and autoimmune diseases such as multiple sclerosis. Conversely, macrophages can also adopt an anti-inflammatory phenotype, producing anti-inflammatory mediators that potentiate tissue regeneration and repair. IL-10 is a known inducer of this anti-inflammatory phenotype. The aim of this study is to interrogate the genetic changes in macrophages induced by IL-10, and to decipher mechanisms that promote an anti-inflammatory state. This will aid the rationale for harnessing macrophages therapeutically and help elucidate their role in disease processes. Murine bone-marrow derived macrophages were harvested and treated with LPS and IL-10. Following 24 hour exposure, an Affymetrix array was conducted. This project involved systematically analysing the array to identify each gene, and completing a literature search using PubMed to interrogate each gene’s potential relevance to four topics related to macrophage function – IL-10 signalling, mitochondrial metabolism, glucose metabolism and multiple sclerosis. Analysis of the array showed 132 genes were significantly upregulated, whereas 142 were significantly downregulated upon IL-10 exposure versus control macrophages. Following exclusion of duplicates, 97 unique entries were identified. Of these, 85 genes (88%) had human homologs, and 94 genes (97%) were protein coding. Following systematic literature review, 40 genes yielded no relevant publications. Following review, seven genes of the remaining 57 had relevant publications across the four topics; namely IgG receptor Fcgr2b, membrane glycoprotein Neuroregulin1, transcription factors Nfil3 and Bhlehe40, metalloprotease Steap4, hormone Adrenomedullin and the enzyme Arginase2. This research serves as a reference study identifying key genes associated with the anti-inflammatory macrophage phenotype following IL-10 stimulation. The seven genes of interest highlight key mechanisms through which the macrophage orchestrates this essential function, and represent potential areas for future therapeutic benefit.

#### P32. Investigating the Impact of COVID-19 on Breastfeeding Rates and Support Structures at Michael Garron Hospital

##### Desiree D'Souza^1^, Jennifer Bordin^2,^, Rebecca S.Y. Tyli^3^, Soraya Visram^4^, Melissa Tai^5^

###### **Correspondence:** Desiree D'Souza

####### ^1^Graduate Entry Medicine, Royal College of Surgeons in Ireland, Dublin, Ireland; ^2^Michael Garron Hospital, Toronto East Health Network, Toronto, Canada; ^3^Health Science Program, McMaster University, Hamilton, Canada; ^4^Michael Garron Hospital, Toronto East Health Network; University of Toronto, Toronto, Canada; ^5^Michael Garron Hospital, Toronto East Health Network; University of Toronto, Toronto, Canada

**Introduction**

The Breastfeeding Clinic at Michael Garron Hospital plays a pivotal role in maintaining the hospital’s “Breastfeeding Friendly” designation by the WHO. The pandemic forced closure of the clinic from mid-March to mid-June 2020. In-hospital lactation consultations were also limited. We assessed the impact of closure of the breastfeeding clinic on breastfeeding rates, and sought to identify the main challenges to breastfeeding in mothers who were confirmed or suspected cases of COVID-19.

**Methods**

We used an explanatory sequential mixed-method approach. Breastfeeding rates from March-May 2019 were compared to 2020. This was followed by a retrospective telephone service evaluation of 3 mothers who tested positive for COVID-19 (100% response rate), and 18 who were under investigation (42% response rate). A reflexive thematic analysis of qualitative data was conducted to identify the barriers and facilitators to breastfeeding.

**Results**

There were no significant differences in the breastfeeding initiation rates. However, the adjusted breastfeeding rates, which account for infants who received one feed other than human milk for a documented medical reason, were significantly lower for March, April, and May 2020. In March and April 2020, 95% of mothers intended to breastfeed, and 91% were provided with breastfeeding education, yet the adjusted breastfeeding rate was 65%. The retrospective telephone survey identified anxiety about the virus, reduced lactation consultation, and isolation as major barriers to breastfeeding. Facilitators included video lactation consultation, and increased time with the baby.

**Discussion**

While breastfeeding initiation was successful, continuity was impaired, potentially due to reduced support postpartum as a result of lockdown. The pandemic has presented compounding difficulties to breastfeeding. This work enabled us to learn directly from patients about their experience, prepare for similar situations, and broaden access to lactation services.

#### P33. Is it possible to predict effects of metabolic surgery?

##### Izabela Karpińska^1^, Joanna Choma^1^, Alicja Dudek^1^, Piotr Małczak^1^, Magdalena Szopa^2^, Piotr Major^1^

###### **Correspondence:** Izabela Karpińska

####### ^1^2nd Department of General Surgery, Jagiellonian University Medical College, Krakow, Poland; ^2^Department of Metabolic Diseases, Jagiellonian University Medical College, Kraków, Poland

**Introduction**

Bariatric surgery was proven to be the most efficient treatment of obesity and type 2 diabetes mellitus (T2DM). Despite detailed qualification, not every patient achieved desirable outcome of T2DM remission after intervention. Recently, DiaBetter and Robert’s scores have been developed to predict diabetes remission after bariatric surgery.

**Aim**

To validate and compare the performance of DiaBetter and Robert’s scores as the predictors of diabetes remission 1 year after surgical treatment.

**Material and methods**

The retrospective analysis included consecutive patients with T2DM who underwent Roux-en-Y gastric bypass (RYGB) or sleeve gastrectomy (SG) between 2009 and 2017 in a single tertiary referral center and completed 1-year follow-up. The DiaBetter and Robert’s scores were calculated for each patient. Each score relationship with diabetes remission was assessed using logistic regression. Discrimination was evaluated by area under the receiver operating characteristic (AUROC) whereas calibration by Hosmer–Lemeshow test.

**Results**

Out of 252 patients enrolled in our study 150 (59.5%) were women whereas 102 (40.5%) were men with median age 48 years. 46.83% of patients underwent SG whereas 53.17% of them had RYGB. The T2D remission rate reached 90.5%. Median of preoperative A1c was 6.75% and preoperative BMI was 45.39 kg/m2, both decreased to 5.8% and 33.09 kg/m2 respectively after 1 year. %EWL after surgery amounted to 53.4%. Either DiaBetter or Robert’s score were predictive of diabetes remission in a logistic regression analysis (OR 0.51; p<0.0001 and OR 1.93; p=0.0031, respectively). The DiaBetter score presented excellent discrimination power (AUROC 0.81; p<0.0001) whereas Robert’s score had poor discrimination with AUROC=0.67 (p<0.0001). Both scores demonstrated statistically good calibration.

**Conclusions**

Both DiaBetter and Robert’s scores can be used in preoperative assessment of diabetes remission after bariatric surgery. DiaBetter score seems to be more accurate than DiaRem score in predicting metabolic outcomes after bariatric surgery.

#### P34. Operating Theatre Practices, Microbes in Air and Post-Operative Infection

##### Sultan Abukhodair^1^, Josh De Marchi^2^, Anna Heaney^2^, Trudi Nelson^3^, Aoife D. Kearney^4^, Abeeda Butt^2^, Hilary Humphreys^4^, Arnold Hill^1,2^

###### **Correspondence:** Sultan Abukhodair

####### ^1^Royal College of Surgeons in Ireland, Dublin, Ireland; ^2^Department of Surgery, Beaumont Hospital, Dublin, Ireland; ^3^Beaumont Hospital, Dublin, Ireland; ^4^Department of Clinical Microbiology, Royal College of Surgeons in Ireland, Education and Research Centre, Beaumont Hospital, Dublin, Ireland

**Introduction**

Surgical site infections (SSI) account for 20% of all nosocomial infections. Intra-operative infection prevention and control (IPC) measures including adherence to aseptic technique. We observed adherence to IPC practices by healthcare workers during surgical procedures and sampled the air for airborne bacteria and particles.

**Methods**

General surgical procedures were audited for compliance with IPC measures (including appropriate use of personal protective equipment and antibiotic prophylaxis) over three weeks. For six procedures, air sampling was with the AES Chemunes Sampl’air Lite Air Sampler and Columbia blood agar plates were incubated for 24 h. For four procedures, airborne particles were also measured using a ParticleScan ProTM Airborn Particle Counter. Patients were followed up in the outpatients or by telephone seven days post-procedure.

**Results**

A total of 31 operations were observed; 25/31 (80.6%) were clean wounds, 3/31 (9.6%) clean-contaminated, 1/31 (3.2%) contaminated, and 2/31 (6.4%) infected. 100% of healthcare workers wore hats, 70.6% wore masks and 50.5% wore scrubs. 22 (70.9%) patients received appropriate prophylaxis, 2 (6.4%) received pre- and intra-operative prophylaxis and 7 (22.6%) did not receive prophylaxis. Airborne bacterial counts were 12-74 (CFU)/m^3^ with a mean of 34 (CFU)/m^3^. Colony counts to the number of people in St Joseph’s were high compared to Beaumont. Airborne particle counts of >1μm were 25-146 with a mean of 75.5 and >5μm were 0-10 with a mean of 4.4. Of 26/31 available for follow up, there were no SSI.

**Discussion**

Most procedures were low risk for SSI, hence there were no SSI. Compliance with IPC was generally good but a larger study is required to correlate SSI, IPC and bacterial/particle counts.

#### P35. Patient Experiences with a Remote Monitoring Pathway for COVID-19

##### Courtney Cheng^1^, Lora Appel^2^, Andrea Scrivener^3^, Christopher Smith^3^

###### **Correspondence:** Courtney Cheng

####### ^1^School of Medicine, Royal College of Surgeons in Ireland, Dublin, Ireland; ^2^OpenLab, University Health Network, Toronto, Canada; ^3^Michael Garron Hospital, Toronto East Health Network, Toronto, Canada

**Introduction**

In response to the COVID-19 outbreak, Michael Garron Hospital developed the CovidCare remote monitoring pathway to provide timely clinical evaluation and management for patients with suspected or confirmed COVID-19. Remote monitoring is increasingly used, but limited data exist on patients’ experiences with these pathways for managing COVID-19. This study aims to describe patients’ experiences with CovidCare, specifically two patient populations: those with medium- or high-level alerts that A) did not return to the emergency department (ED) and were successfully managed at home, and B) those who returned to ED but were not admitted.

**Methods**

Semi-structured phone interviews were conducted, transcribed, and analysed using grounded theory.

**Results**

Across 35 interviews (response rate of 66%), three main themes were identified: the program provided emotional support (a sense of security, reduced feelings of depression and loneliness, and decreased fear and anxiety); was informative (taught patients COVID-19-related precautions, instructed patients on how to self-monitor COVID-19 symptoms, and informed patients about self-care when coping with COVID-19), and motivated patients to self-monitor and self-manage (facilitated self-management, prompted self-management, and encouraged self-monitoring). Patients in both groups also reported nurses at times urging them to go to ED despite feeling able to manage at home, and only a few patients in Group B returned to ED for issues directly related to COVID-19.

**Discussion**

The CovidCare pathway was well-received by most interviewed patients. Both groups identified the tendency for nurses to recommend ED assessment for worsening symptoms; however, only a few patients in Group B returned to ED for issues directly related to COVID-19, limiting further analysis into why this advice may have affected them differently. Further research should explore the tendency of the CovidCare pathway to recommend ED assessment to improve its efficiency and applicability for other remote monitoring programs.

#### P36. Patterns of Metastatic Disease in Stage IV ALK-rearranged Non-Small Cell Lung Cancer Patients

##### Simren Chotai^1^, Karmugi Balaratnam^2^, Luna Jia Zhan^2^, Devalben Patel^2^, Wei Xu^2^, Catherine Brown^2^, Katrina Hueniken^2^, Geoffrey Liu^2^

###### **Correspondence:** Simren Chotai

####### ^1^Royal College of Surgeons in Ireland, Dublin, Ireland; ^2^Princess Margaret Cancer Centre, Toronto, Canada

**Introduction**

Anaplastic Lymphoma Kinase (ALK) gene rearrangements occur in 2-4% of Non-Small-Cell-Lung-Cancers (NSCLC). This study aims to determine the patterns of metastatic disease in these rare Stage IV ALK-rearranged NSCLC Patients, as there is limited real-world data on this topic.

**Methods**

In a cohort of Stage IV ALK-rearranged NSCLC patients from Princess Margaret Cancer Centre, a comprehensive Canadian Cancer Centre, manual data ion was performed from the electronic patient records, supplemented with patient-reported demographic survey data. Descriptive summary statistics were performed.

**Results**

Of 105 Stage IV ALK-rearranged NSCLC patients, 54% were female. The median (range) age was 60 (31-92) years; 31% were Caucasian; 40% were Asian; 75% were lifetime never-smokers; 75% were de novo Stage IV. The median (range) number of metastatic sites was 1 (1-6) at baseline, and after 1 year, 2 (1-10) sites. At baseline, 53% of patients had 1 metastatic site; 27% had 2 sites; while 10% had 3 sites, and 10% had 4-6 sites. From baseline to 1 year after baseline, the proportion of patients with bone metastases had risen from 32% to 43%; for brain, 25% to 37%; for liver, 11% to 14%; for lung, 17% to 28%; for adrenal, 6% to 11%; and for pleural-pericardial disease, 44% to 60%. Some ALK-rearranged NSCLC patients exhibited unusual areas of metastases, including leptomeningeal, choroidal, kidney, peritoneum, pancreas and adnexa.

**Discussion**

Compared with historical local cohorts of general NSCLC patients, ALK+ NSCLC patients were younger, more likely to be lifetime never-smokers and of Asian descent, with greater prevalence of pleural-pericardial disease, bone and brain metastases and presenting with some unusual patterns of metastatic spread. Stage IV ALK+ NSCLCs form a distinct clinical and demographic patient group, when compared to the general NSCLC population and evaluating the metastatic patterns can provide a better understanding about the disease progression.

#### P37. Psammocarcinoma of the Ovary: a Systematic Review

##### Ilia Mihaylov^1^, Diana Strateva^2^, Angel Yordanov^2^

###### **Correspondence:** Ilia Mihaylov

####### ^1^Medical University Pleven, Pleven, Bulgaria; ^2^Department of Gynecologic Oncology, Medical University Pleven, Pleven, Bulgaria

Psammocarcinoma is a rare subtype of serous epithelian neoplasms. In the literature it most commonly described in the ovaries and the peritoneum. It is characterized by massive psammoma body formation, low grade cytologic features, and invasiveness. Its clinical behavior is similar to serous borderline tumors.

We performed a search in PubMed and ScienceDirect with the following terms: “ovary, psammocarcinoma” and “ovarian cancer, psammocarcinoma”. A total of 211 results came back before the beginning of October 2020. We excluded all studies and reports for other cancer localization, type and duplicating articles. We paid special attention to the patient’s: age, cancer staging, type of surgery, chemotherapy, recurrence.

After a careful evaluation of the results 44 cases of psammocarcinoma of the ovary have been discovered. The median age for them is 52.8 years (19-73). The most common stage by FIGO is IIIB (in 11 patients). Stage III is the most common – 24 of the cases while there are 17 cases with unknown staging. The surgical interventions differ in many cases as the age, stage of the cancer and the circumstances differ. However, the most common is radical hysterectomy with bilateral salphingo-oophorectomy, omentectomy. In some cases, taking biopsy from the peritoneum and lymph node dissection were performed. There are cases of debulking in order to protect the natal capabilities. The most common adjuvant chemotherapy includes Carbapenem and Paclitaxel (PC therapy) – 7 patients. One female underwent neoadjuvant PC chemotherapy. Finally, 7 out of the 24 patients with follow-up either died of the disease or had recurrence.

Ovarian psammocarcinoma is a rare and aggressive oncological condition. Of the total of 44 patients, found in PubMed and ScienceDirect.com there were many instances of poor description of the case itself. Yet, the late diagnosing of the disease and its aggressive nature are a fact.

#### P38. Radiomics analysis of PET / CT findings with fludeoxyglucose (18F) as a biomarker in the differentiation of spondylodiscitis and bone metastasis in patients with focal lesions in the spine

##### Natasa Brisudova^1^, Sona Balogova^2^, Iveta Waczulikova^1^

###### **Correspondence:** Natasa Brisudova

####### ^1^Faculty of Mathematics, Physics and Informatics, Charles University, Bratislava; ^2^Faculty of Medicine, Charles University, Bratislava

**Introduction**

Early initiation of targeted treatment can prevent possible irreversible neurological complications of spondylodiscitis (SD) and / or spinal metastases (MET), but differentiation may be a diagnostic problem, especially in the early stages. **Aim:** Identify the radiometric characteristics of PET with FDG helping to distinguish SD and MET.

**Methods**

Retrospective analysis of 31 second- and higher-order radiometric elements in 60 patients with confirmed SD (n = 30) and MET of various malignancies (n = 30). A total of 40 SD findings and 40 MET findings were analyzed using LIFEx freeware, which allows the calculation of conventional, textural and shape elements of diagnostic images. The clinical characteristics of the patients (non-parametric Wilcoxon rank sum test) were compared using the statistical software RStudio and their acceptable diagnostic accuracy was tested using the ROC curve. Furthermore, the predictive ability to distinguish SD and MET was tested using machine learning, where three methods were tested (multiple logistic regression, random forest and support vector machines), with three different ways of selecting training and test data (K-fold cross-validation, Leave- One-Out Cross-Validation, Train test split).

**Results**

When SD and MET were distinguished, 24/31 radiometric elements were confirmed as statistically significant (p <.05) and in 9/24 the AUC was> 80% for diagnostic accuracy. The highest values ​​were reached by the parameters GLZLM_ZP (cut-off = 0.38, AUC = 83.25%), NGLDM_Contrast (cut-off = 0.17, AUC = 84.7%) and GLRLM_GLNU (cut-off = 46.1, AUC = 88.8%). In machine learning, the most effective method was Random Forest (cut-off = 0.28, AUC = 98.61%) with the method of data selection Train test split.

**Conclusions**

The results confirm radiomatic analysis and machine learning as a possible direction in distinguishing SD and MET in PET / CT with FDG and support their further validation.

#### P39. Renin-Angiotensin-Aldosterone System as a Target for Facilitating Sinus Rhythm Maintenance after Electrical Cardioversion in Hypertensive Atrial Fibrillation Patients

##### Baiba Kokina^1^, Oskars Kalejs^2^

###### **Correspondence:** Baiba Kokina

####### ^1^Faculty of Medicine, Riga Stradins University, Riga, Latvia; ^2^Department of Internal Diseases, Riga Stradins University; Latvian Centre of Cardiology, Pauls Stradins Clinical University Hospital, Riga, Latvia

**Introduction**

Sinus rhythm maintenance after electrical cardioversion (ECV) remains challenging in comorbid atrial fibrillation (AF) patients, arterial hypertension (AH) having established role, both pathophysiologically linked by pressor effects and renin-angiotensin-aldosterone system (RAAS) upregulation. Non-antiarrhythmic drug (non-AAD) upstream therapies, including RAAS inhibition by angiotensin-converting enzyme inhibitors (ACEIs)/angiotensin receptor blockers (ARBs) and mineralocorticoid receptor antagonists (MRAs), come into focus, showing heterogeneous results. This study evaluates, whether and how convincing RAAS inhibition, by ACEIs/ARBs and combination with MRAs, can add benefit to AAD therapy, facilitating sinus rhythm maintenance after ECV in AF patients with pharmacologically controlled AH.

**Methods**

The study was conducted among AF patients after successful ECV in Latvian Centre of Cardiology. Additional inclusion criteria were pharmacologically controlled AH and class IC or III AAD prescription, not restricting specific medication intake. After baseline interview, 1-, 3-, 6-, 9-, 12-month follow-up was conducted. Medication effectiveness was evaluated using MS Excel and SPSS Statistics, calculating odds ratios (ORs) and 95% confidence intervals (CIs), with significance level α=0.05.

**Results**

99 patients were included. Among participants not using any RAAS inhibitor (20.2%), AF recurrence rate comprised 65.0%. Present RAAS inhibitor (79.8%) therapy demonstrated recurrence rate 48.1%, with 53.4% and 33.3% relapses for ACEI/ARB and concomitant ACEI/ARB and MRA intake, respectively. Compared to non-use, presence of RAAS inhibitor reduced OR for AF recurrence by 50.1% (OR 0.499, 95%CI 0.180-1.383, p=0.181), ACEI/ARB intake by 38.2% (OR 0.618, 95%CI 0.216-1.773, p=0.371), combined ACEI/ARB and MRA use by 73.1% (OR 0.269, 95%CI 0.074-0.979, p=0.046).

**Discussion**

RAAS inhibition demonstrated therapeutic potential adjunctive to AADs, showing improved sinus rhythm maintenance tendency in pharmacologically controlled hypertensive AF patients, with statistically significant effect demonstrated by concomitant ACEI/ARB and MRA intake. Highlighting effects beyond blood pressure reduction, findings are supportive of MRA use in AF patients with AH, yet not primarily emphasized for this group.

#### P40. Tamoxifen and doxorubicin hydrochloride chemotherapy: in vivo efficacy of different doses, formulations, and treatment schemes

##### Vityala Yethindra^1^, Tugolbai Tagaev^2^, Cholpon Dzhumakova^3^, Asel Namazbekova^4^

###### **Correspondence:** Vityala Yethindra

####### ^1^International Higher School of Medicine, International University of Kyrgyzstan, Bishkek, Kyrgyzstan; ^2^Department of Public Health and Healthcare, I.K. Akhunbaev Kyrgyz State Medical Academy, Bishkek, Kyrgyzstan; ^3^Department of Gastroenterology, National Center of Oncology and Hematology, Bishkek, Kyrgyzstan, ^4^Department of Cancer registry, National Center of Oncology and Hematology, Bishkek, Kyrgyzstan

**Introduction**

The use of combination therapy in cancer treatment helps to decrease the treatment doses, and consequently reduces non-specific cytotoxicity and resistance. We aimed to investigate whether liposomal and non-liposomal doxorubicin (DOX), alone or in combination with tamoxifen (TAM), could exhibit antitumor effects.

**Methods**

We assessed the efficacy of various anticancer treatment regimens against the development of Walker 256 tumors in white rats (32 animals weighing 250–300 g). DOX (5 mg/kg) was administered, either non-formulated or formulated in liposomes, alone or in combination with TAM (0.5 mg/kg), intraperitoneally in the following two different regimens: prophylaxis (once daily for 15 days) + treatment (once daily for 5 days), and treatment only (once daily for 5 days). The prophylaxis + treatment regimen was also used in the context of combination therapy with a lower dose of TAM (0.25 mg/kg) and a shorter treatment period (3 days). Tumor growth was recorded. Data are presented as mean ± standard deviation. The Student’s *t*-test was used for two-group comparisons; p<0.05 was considered significant.

**Results**

The administration of liposomal DOX, 5 days after tumor inoculation, led to a significant inhibition of tumor growth, 17 days after the start of the experiment; 43% *versus* the control group. Noteworthy, tumor growth suppression was similar in the context of DOX monotherapy (in liposomes) and combination therapy. Further, the combination of liposomal DOX and TAM was the most effective approach for suppressing tumor growth (via prophylactic + treatment regimen); a significant difference in the tumor growth curves was observed from day 5, with a growth inhibition of 32%.

**Discussion**

The most effective anticancer approach was the administration of liposomal DOX in combination with TAM in a prophylactic + therapeutic regimen, which provided considerable safety and a significant inhibition of tumor growth.

#### P41. The association between Maternal Micronutrients Supplementation and child birthweight in Sa’ad Abualela hospital and Suba teaching hospital, Khartoum, Sudan in 2019

##### Arwa Alsharief^1^, Heitham Awadalla^2^

###### **Correspondence:** Arwa Alsharief

####### ^1^University of Khartoum, faculty of medicine, Khartoum, Sudan; ^2^University of Khartoum, faculty of medicine, department of Community medicine,Khartoum, Sudan.

**Introduction**

Birth weight is a strong predictor for child health and development and is influenced by many factors including maternal health. Micronutrients supplementation during pregnancy is beneficial in improving the maternal health and maybe of potential benefit to fetal outcomes. This study aims to assess the effect of antenatal multiple micronutrients supplements on infant birth weight compared with the routine iron-folate supplements.

**Methods**

This was a cross- sectional study of 223 women with no chronic illnesses conducted in Sa’ad Abualela and Suba university hospitals, Khartoum, Sudan. Data were collected through structured interviews and the birth weights were recorded as measured by midwives .women were asked about their socio-demographic data and health factors. Their height and weight were measured to classify their BMI. Information about their antenatal micronutrients intake status, type of supplements and duration of intake were also collected. Data were analysed using SPSS 21.

**Results**

Of 223 women participated in this study 13.6 % of live born children were of low birth weight. Mean 2.89 ± 0.56 .The proportion of low birth weight was higher in female than male infants. A total of 68.78% mothers consumed multiple micronutrients (n=152), while 20.81% were on the iron-folate supplements(n=46). There was a small non-significant increase in birth weight in the mothers receiving multiple micronutrients compared with the ones receiving iron–folic acid supplements (2.94 ± 0.56 vs. 2.86 ± 0.44 kg ) respectively. (p=0 .25) . No additional benefit was elicited of multiple micronutrients in reducing the risk of low birth weight which was surprisingly higher in this group (13.8%) than the iron-folate group(13.1%).

**Conclusion**

In Sudan, Multiple micronutrients confers no additional benefit over iron-folic acid supplements in reducing the risk of low birth weight. in relatively healthy mothers. However, they resulted in non-statistically significant increase in the mean birth weight.

#### P42. Utilization patterns of drugs used in the treatment of rheumatoid arthritis in a tertiary care hospital in Oman

##### Moosa Allawati^1^, Sanad Al-Malki^1^, Aly Abdelrahman^2^

###### **Correspondence:** Moosa Allawati

####### ^1^College of Medicine and Health Sciences, Sultan Qaboos University, Seeb, Oman; ^2^Department of Pharmacology and Clinical Pharmacy, College of Medicine and Health Sciences, Sultan Qaboos University, Seeb, Oman

**Introduction**

Rheumatoid arthritis (RA) is an autoimmune rheumatic disease (ARD) in which different synovial joints are affected. Patients are prescribed anti-rheumatic agents for symptomatic treatment and/or to delay the progression of the disease. Those agents include glucocorticoids, disease-modifying antirheumatic drugs (DMARDs), biological medications and non-steroidal anti-inflammatory drugs (NSAIDs). The aim of the study was to identify the utilization patterns of glucocorticoids and DMARDs prescribed to RA patients who visited Sultan Qaboos University Hospital (SQUH).

**Methods**

A retrospective study was conducted in which the data of 200 RA patients who visited SQUH's rheumatology clinic were noted down. The data included patients' data besides the antirheumatic agents that were prescribed in their last one to two visits. The data was accessed using the hospital’s system. We noted down all types of data and variables collected from their records such as the doses, routes of administration and the general prescription patterns according to age and gender. Data were analyzed using SPSSv23 for any important statistical associations.

**Results**

Out of 200 RA patients (179 females and 21 males), 37.0% were prescribed two medications. More than three-quarters of the patients (77.0%) were prescribed DMARDs while glucocorticoids were prescribed to 49.5% of the patients, and both classes of drugs were more commonly prescribed to the oldest age group (>= 65 years old). Most of the patients (89.5%) were prescribed oral medications.

**Discussion**

The data in this study showed that there were differences in the prescription patterns of antirheumatic drugs among the genders and age groups. Methotrexate was the most commonly prescribed medication which was in accordance with previous studies. The study would help doctors know the utilization patterns of antirheumatic drugs but more work is needed regarding this topic in Oman as the current researches are few and outdated.

